# Applications of gut microbiota in patients with hematopoietic stem-cell transplantation

**DOI:** 10.1186/s40164-020-00194-y

**Published:** 2020-12-04

**Authors:** Jifeng Yu, Hao Sun, Weijie Cao, Lijie Han, Yongping Song, Dingming Wan, Zhongxing Jiang

**Affiliations:** 1 Department of Hematology, The First Affiliated Hospital of Zhengzhou University, Zhengzhou, 450052 China; 2 Academy of Medical and Pharmaceutical Sciences of Zhengzhou University, Zhengzhou, 450052 China; 3 Department of Radiotherapy, The First Affiliated Hospital of Zhengzhou University, Zhengzhou, 450052 China; 4 The Affiliated Cancer Hospital of Zhengzhou University and Henan Cancer Hospital, Zhengzhou, 450008 China

**Keywords:** Targeted modulation therapy, Gut microbiota, Hematopoietic stem cell transplantation (HSCT)

## Abstract

Studies of the gut microbiota (GM) have demonstrated the close link between human wellness and intestinal commensal bacteria, which mediate development of the host immune system. The dysbiosis, a disruption of the microbiome natural balance, can cause serious health problems. Patients undergoing allogeneic hematopoietic stem cell transplantation (allo-HSCT) may cause significant changes in GM due to their underlying malignancies and exposure to extensive chemotherapy and systemic antibiotics, which may lead to different disorders. There are complex and multi-directional interactions among intestinal inflammation, GM and immune reactivity after HSCT. There is considerable effect of the human intestinal microbiome on clinical course following HSCT. Some bacteria in the intestinal ecosystem may be potential biomarkers or therapeutic targets for preventing relapse and improving survival rate after HSCT. Microbiota can be used as predictor of mortality in allo-HSCT. Two different strategies with targeted modulation of GM, preemptive and therapeutic, have been used for preventing or treating GM dysbiosis in patients with HSCT. Preemptive strategies include enteral nutrition (EN), prebiotic, probiotic, fecal microbiota transplantation (FMT) and antibiotic strategies, while therapeutic strategies include FMT, probiotic and lactoferrine usages. In this review, we summarize the advance of therapies targeting GM in patients with HSCT.

## Introduction

Thousands of different species of micobiome are colonized at different sites of human body and play a key role in maintaining our health or promoting disease [1]. Studies of the gut microbiota (GM) have demonstrated the close link between human wellness and intestinal commensal bacteria, which mediate development of the host immune system [2]. The dysbiosis, a disruption of the microbiome natural balance, can cause serious health problems [3, 4]. Patients undergoing allogeneic hematopoietic stem cell transplantation (allo-HSCT) may cause significant changes in GM due to their underlying malignancies and exposure to extensive chemotherapy and systemic antibiotics, which may lead to biological disorders. Studies have shown that there are complex and multi-directional interactions among intestinal inflammation, GM and immune reactivity after HSCT. There is mounting evidence for the considerable effect of the human intestinal microbiome on clinical course following HSCT [5–7]. The abundance or presence of some bacteria in the intestinal ecosystem may be potential biomarkers or therapeutic targets for preventing relapse and improving survival rate after HSCT [8]. Most recent study showed that microbiota can be used as predictor of mortality in allo-HSCT [9]. Many studies have showed that targeted modulation of GM in patients with HSCT has potential therapeutic implications [10]. In this review, we summarize the advance of therapies targeting GM in patients with HSCT.

## Mechanism of gut microbiota in graft versus host disease (GvHD)

Gut GvHD is the result of conditioning toxicity and immune activation associated with injury of the stem-cell compartments along with Paneth and goblet cells in the intestinal mucosa [11]. This leads to increased intestinal permeability, inflammation, and reduction of the mucous membrane [12, 13]. The mucus layer produced by goblet cells acts as a physical barrier in the gut and regulates the relationship between the microbiota and the host [14]. Intestinal epithelial cells, dendritic cells, and macrophages express pattern recognition receptors, such as Toll-like receptors, which can recognize microbe-associated molecular patterns. Activation of these receptors triggers proinflammatory cytokine response and presents antigens to regulatory T cells (Tregs). Activation of Tregs conveys tolerance towards commensal bacteria [14]. Gut bacteria produce Butyrate and other short-chain fatty acid (SCFA), which exert anti-inflammatory effects on the macrophages and the dendritic cells through inhibiting histone deacetylase (HDAC), inhibiting NF-κB signaling and increasing IL-10 expression [15]. Metabolomics analysis of human acute graft-versus-host disease reveals changes in host and microbiota-derived metabolites [16]. Most recent study revealed that Butyrate was significantly decreased in all gastro-intestinal (GI) acute GvHD (aGVHD) stages. Specific microbiota and metabolic alterations were associated with aGVHD severity and may be useful for diagnostic and pathophysiologic purposes [17].

Segmented filamentous bacteria can penetrate the mucus layer and interact with epithelial cells, inducing the differentiation of T helper 17 (Th17) cells [18]. Th17 cells are specialized in responses to extracellular bacteria and fungi by secretion of cytokines such as IL-17A, IL-17F, IL-21, and IL-22 [18]. The cytokines produced by Th17 cells induce secretion of antimicrobial peptides such as the α-defensins and RegIIIγ by the Paneth cells [14]. Patients with hematological diseases requiring HSCT undergo extensive preconditioning chemotherapy as well as antibiotic or antifungal treatments. Although antibiotic treatment in HSCT patients is essential in many patients to avoid bacterial infections, these interventions result in the disruption of the gut microbiota and its equilibrium and can cause additional gastrointestinal damage. Studies have shown that there is a high risk of bacterial infection during transplantation, and subsequent GvHD and low GM diversity are closely associated with transplant related mortality [19, 20]. Alteration of the intestinal microbiota by broad-spectrum antibiotic use correlates with the occurrence of intestinal GvHD [21]. GvHD occurs in a large number of patients receiving allo-HSCT, resulting in a mortality rate of up to 30% [22].

Extensive studies of monitoring microbiome alterations have been done, especially regarding the GM and the GvHD in patients with HSCT [7–10, 23]. The most recent study showed patterns of microbiota disruption during allo-HSCT were similar across transplantation centers and geographic locations. Patterns were characterized by loss of diversity and domination by single taxa. Higher diversity of intestinal microbiota at the time of neutrophil engraftment was associated with lower mortality [9]. The microbiota can be used as predictor of mortality in allo-HSCT [10], and the constitution of the intestinal microbiota at neutrophil engraftment and GM score can predict the development of aGvHD following myeloablative allo-HSCT [24, 25]. These results supported the idea for modulation of the GM in patients with HSCT.

## Modulation of the GM in HSCT

**Fig. 1:**
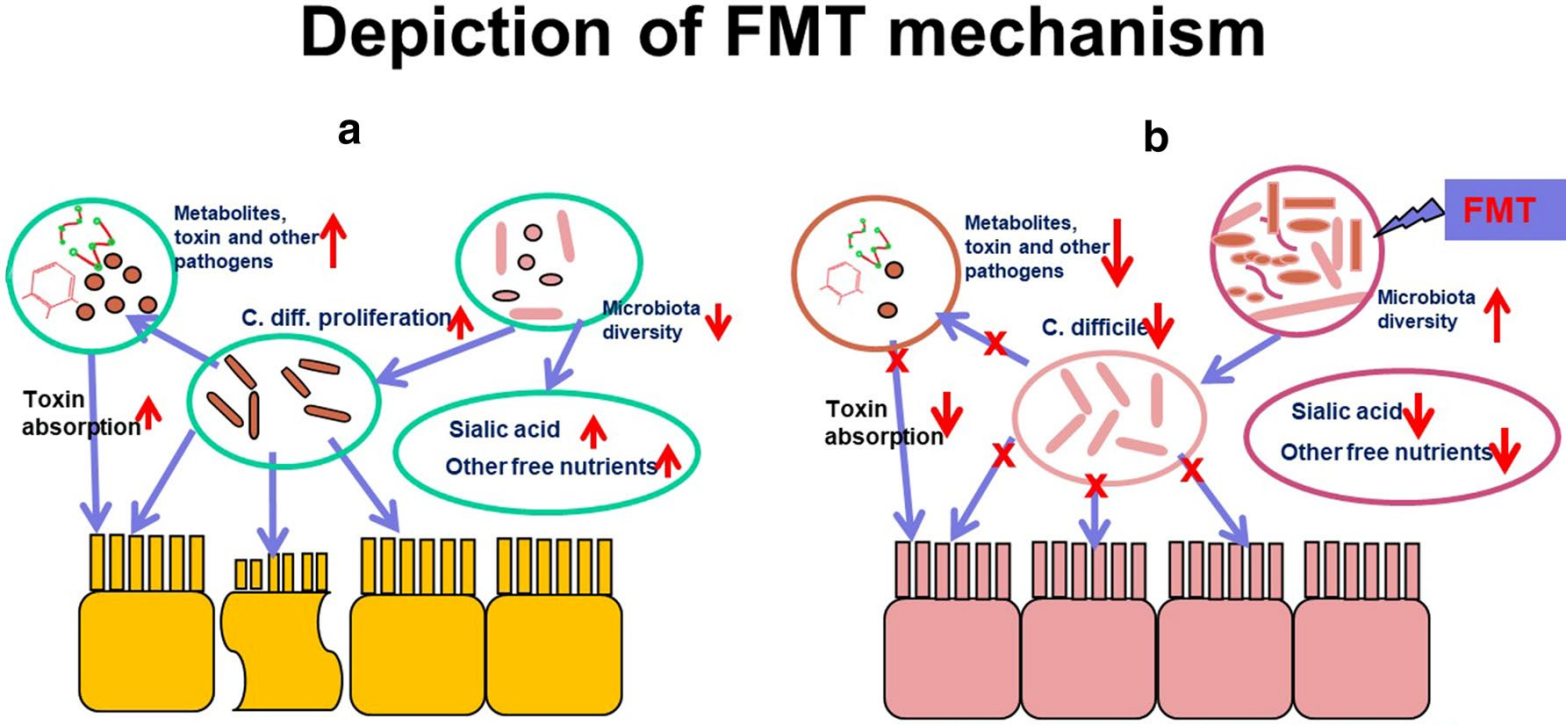
Depiction of FMT mechanism. **a** HSCT and related procedures caused toxin secretion and intestinal epithelial cells damage. **b** FMT treatment made the microbiota diversity and intestinal epithelial cells recovery

After the confirmation of the strong impact of the GM on all aspects of HSCT, modulating GM composition in order to improve clinical outcomes has been proposed following different clinical trials. The main interventions described in the literature included two different potential strategies, preemptive and therapeutic, both of which have been used for preventing or treating GM dysbiosis during HSCT. Preemptive strategies include enteral nutrition (EN), prebiotic, probiotic, fecal microbiota transplantation (FMT) and antibiotic strategies, while therapeutic strategies include FMT, probiotic and lactoferrine usages [10] (Fig. 1).

## Nutritional supplementation in HSCT

Nutrition support for patients includes two different kinds of options, EN and parenteral nutrition (PN). EN is a kind of nutritional support that provides nutrients and other nutrients needed by metabolism through the gastrointestinal tract. Meanwhile, PN is through intravenous injection into the blood circulation to supplement nutrition.

Many studies have confirmed the connection between nutrition and the human microbiome in maintaining human health [26–30]. Traditionally, the first nutritional approach in post-HSCT patients is parenteral nutrition (PN), which is associated with several clinical adverse effects, supporting EN as a preferential alternative. The effect of PN and starvation on the intestinal ecosystem during HSCT has been studied and the results showed the decreased microbial richness and diversity [31, 32]. PN has been associated with the loss of commensal bacteria belonging to the genus Blautia and induces gut mucosal atrophy, promoting bacterial translocation and altering SCFA production [33–36]. Clinical data show that EN is associated with better outcomes in terms of survival, infection, and aGvHD [37–40]. Recent studies have showed that EN is a feasible and nutritionally adequate method of nutritional support for children undergoing allo-HSCT. EN can protect children undergoing allo-HSCT from blood stream infections [41] and promote the recovery of gut microbiome homeostasis [42]. In patients with EN, structural and functional probiotic GM distribution is rapidly restored after HSCT, which may reduce the risk of systemic infection and GvHD onset [42]. However, more studies are needed to further explore the role of the type of nutritional support in preserving GM during HSCT. These nutritional supports include applications of prebiotics, antibiotics, lactoferrin and probiotics.

## Prebiotics

The first commonly used nutrition support option is prebiotics. Prebiotics are defined as ‘a substrate which is selectively utilized by host microorganisms conferring a health benefit’ [43]. This term usually refers to indigestible carbohydrates, such as dietary fibers, which are fermented in the colon by commensal bacteria to modify the microbiota and produce metabolites with potential immunomodulatory effects [44]. Examples of these fibers include resistant starches, fructooligosaccharides (including inulin), and galacto-oligosaccharides, which are found in a variety of foods including onions, oats, garlic, asparagus, and human milk. Different nutritional strategies have been explored in patients with HSCT in order to modify the GM. Tavil et al. utilized a diet richer in fibre in a patient in the pre-HSCT period, which correlated with earlier neutrophil engraftment and a shorter duration of febrile neutropenia [45]. Preemptive enteral supplementation with glutamine, fiber and oligosaccharide strategy is an effective supportive therapy to decrease the severity of mucosal damage in HSCT [46]. Currently, there is only one ongoing clinical trial using prebiotic to promote a healthy gut microbiome in pediatric HSCT recipients in the United States (NCT04111471).

Prebiotics are metabolized by selected intestinal microorganisms and produce a variety of compounds through fermentation, including SCFAs butyrate, acetate and propionate. Several important studies highlighting the mechanism of prebiotics effect on gut mucosa and host immune response mediated by the intestinal microbiota [47]. SCFAs affect the host by (i) serving as a direct substrate for intestinal epithelial metabolism and maintenance of the mucosal barrier, (ii) affecting immune cell signaling and proliferation, (iii) altering epigenetic modifications, (iv) impacting microbial-microbial and microbial-host interactions, and (v) influencing chemotherapy efficacy and toxicity [47].

Butyrate and related SCFAs are major products of prebiotic metabolism. SCFAs can change the signaling of host immune cells: butyrate in the colonic lumen can increase the proliferation of host Tregs [48] and activate dendritic cells through signaling via chemokine G-protein–coupled receptors [49]. Thus it causes the differentiation of naive T cells into Tregs functions to suppress other immune cells that may induce inflammation.

SCFAs can also act as HDAC inhibitors that change DNA structure and transcription, altering leukocytes and potentially affecting cancer [50] and infection risk [51]. Epigenetic changes have been increasingly implicated in hematologic malignancies like acute myeloid leukemia (AML) and may be affected by the GM. Prebiotics not only impact the risk of infection and GvHD, but also may influence chemotherapy efficacy and toxicity through cellular signaling pathways influenced by SCFA production in patients with cancer [52].

Emerging strategies for prebiotics have been developed in recent years by investigating non-fiber dietary supplements, such as vitamin A, on both microbiota composition and HSCT outcomes [43, 53]. One study showed that vitamin A levels in patients 30 days after HSCT predicted the incidence of GvHD and it may lead to the differentiation of naive T-cells into Tregs rather than Th17 cells, facilitating mucosal tolerance and improving mucosal barrier integrity [54]. Meanwhile, commensal bacteria may inhibit retinoid metabolism in the intestinal epithelium, reduce IL-22 levels and prevent dysbiosis [55]. The increased IL-22 level in children with GI aGvHD further supports the relationship between retinoic acid metabolism, IL-22 level and GvHD [56]. Table 1 summarizes the main on-going studies regarding dietary nutrition in HSCT. Especially, there are one completed (NCT03039257) and two ongoing clinical trials (NCT03202849, NCT03719092) with vitamin A supplementation in patients with HSCT (Table 1), and another ongoing trial directly administering IL-22 Fc as a potential therapy to attenuate GI GvHD (NCT02406651).

**Table 1:** On-going dietary nutrition and HSCT related clinical trials

Clinical trials. gov identifier	Clinical trial title	Phase	Enrollment number	Disease conditions	Status	Lead institution/location
NCT03083327	Prophylactic Early PN in HPT/BMT	Not applicable	408	Hematologic neoplasms	Recruiting	University of Sydney Australasian Bone Marrow Transplant Recipient Registry University of Roma La Sapienza
NCT03534674	Vitamin D3 Supplementation for AlloHSCT-RCT	Not applicable	84	Vitamin D deficiency	Unknown	Vancouver General Hospital Vancouver, British Columbia, Canada
NCT03710031	Developing Self-Management Interventions After HCT	Unknown	55	HSCT	Recruiting	University of Florida
NCT02512718	Safety and Tolerability of Intravenous Fish Oil Lipid Emulsion in Children Undergoing Hematopoietic Cell Transplantation	1	20	HSCT	Recruiting	Boston Children's Hospital Boston, Massachusetts, United States
NCT02763033	Dietary Manipulation of the Microbiome-metabolomic Axis for Mitigating GVHD in Allo HCT Patients	2	70	HSCT	Recruiting	University of Michigan Cancer Center Ann Arbor, Michigan, United States
NCT03016130	Comparing Two Diets in Patients Undergoing HSCT or Remission Induction Chemo for Acute Leukemia and MDS (UF-BMT-LDND-101)	3	470	Leukemia Myelodysplastic syndromes	Recruiting	UF Health Cancer Center Gainesville, Florida, United States
NCT03039257	Vitamin A Replacement in Patients Undergoing HSCT and Its Role on MBI-LCBI Rates	Not applicable	12	HSCT	Completed	Cincinnati Children's Hospital Medical Center Cincinnati, Ohio, United States
NCT03202849	A Randomized Trial of Vitamin D Supplementation With or Without Vitamin A in Stem Cell Transplantation	Not applicable	100	HSCT	Recruiting	Cincinnati Children's Hospital Medical Center Cincinnati, Ohio, United States
NCT03557749	Monitoring of Immune and Microbial Reconstitution in (HCT) and Novel Immunotherapies	Not applicable	1600	Immune and microbial reconstitution Systemic viral infection GvHD	Recruiting	University of Minnesota Masonic Cancer Center Minneapolis, Minnesota, United States
NCT03719092	High Dose Vitamin A in Preventing Gastrointestinal GVHD in Participants Undergoing Donor Stem Cell Transplant	Not applicable	28	Allogeneic HSCT	Not yet recruiting	Ohio State University Comprehensive Cancer Center National Cancer Institute (NCI)
NCT03727113	Optimization of Antibiotic Treatment in Hematopoietic Stem Cell Receptors	Not applicable	180	HSCT GvHD	Recruiting	Virgen del Rocío University Hospital, Seville Sevilla, Seville, Spain
NCT03918343	Lipopolysaccharide Metabolism and Identification of Potential Biomarkers Predictive of Graft-versus-host Disease After Allogeneic Stem Cell Transplantation	Not applicable	98	Hematologic diseases HSCT	Recruiting	Centre Hospitalier Universitaire de Besançon Besançon, France Centre Hospitalier Universitaire de Nancy Nancy, France
NCT04024618	Feasibility Study Comparing Enteral vs Parenteral Nutritional Outcomes in Autologous Stem Cell Transplant Patients	Not applicable	40	Malignant hematologic neoplasm	Recruiting	London Health Sciences Centre-Victoria Hospital London, Ontario, Canada
NCT04146870	Nutritional Status of Patients After Hematopoietic Stem Cell Transplantation	Unknown	200	Nutrition HSCT	Recruiting	The First Affiliated Hospital of Soochow University
NCT04172818	Feasibility Study of a Diary for Allogenic Hematopoietic Stem Cell Transplantation Patients and Families	Not applicable	20	Hematopoietic/lymphoid cancer Psychological disorder	Not yet recruiting	CHU Amiens Amiens, France
NCT04177004	Human Lysozyme Goat Milk for the Prevention of Graft Versus Host Disease in Patients With Blood Cancer Undergoing a Donor Stem Cell Transplant	1	36	Allogeneic HSCT Hematopoietic and lymphoid cell neoplasm	Not yet recruiting	City of Hope Medical Center Duarte, California, United States
NCT02406651	Study of IL-22 IgG2-Fc (F-652) for Subjects With Grade II-IV Lower GI aGVHD	1,2	27	GvHD	Active, not recruiting	City of Hope Duarte, California, United States
NCT04425642	Effects of Parenteral Nutrition in HSCT	Not applicable	120	Hematologic malignancy	Recruiting	Pavlov First Saint-Petersburg State Medical University Saint Petersburg, Russian Federation

## Lactoferrin

Lactoferrin, a glycoprotein of transferrin family, is an iron-binding protein with pleiotropic functions, such as antianemic, antimicrobial, anti-inflammatory, immunoregulatory, antioxidant, and anti-cancer activity, and is also involved in intestinal epithelial regeneration and iron homeostasis [57]. Recently, lactoferrin has been used as new specific molecule in dysbiosis prevention. Lactoferrin and N-terminal peptide-derivatives have been studied in preclinical models, and can reduce bacterial translocation, improving GM eubiosis [58, 59]. Administration of lactoferrin in an HSCT patient showed that symptoms of gut GvHD disappeared soon after lactoferrin therapy was started [60]. Introduction of probiotics with a regimen containing bovine lactoferrin for preterm infants in New Zealand has been associated with significant reductions in necrotizing enterocolitis (NEC) and late onset sepsis [61]. A pooled analysis of individual patient data from two randomized controlled trials demonstrated bovine lactoferrin supplementation protects against late-onset sepsis in infants < 1500 g, especially among infants not receiving human milk [62]. Study by using probiotic and lactoferrin prebiotic were administered in mice showed both Clostridioides difficile inoculation and treatment with vancomycin or fidaxomicin reduced microbiota diversity; however, dysbiosis associated with fidaxomicin was milder than with vancomycin [63]. The use of lactoferrin, or short peptide derivatives that retain the cationic N-terminal moiety that is essential for the anti-microbial and anti-inflammatory activity, may prove to be a promising versatile class of agents for managing the complications that arise from HSCT [64]. Lactoferrin has also been used for many other different clinical applications and has more potential perspectives on its prophylactic and therapeutic applications in the future [57]. Additionally, lactoferrin offers a promising biodegradable well tolerated material that could be exploited both as an active therapeutic and drug nanocarrier. Lactoferrin-based nanocarriers have been demonstrated as efficient platforms for delivery of anti-parkinsonian, anti-Alzheimer, anti-viral drugs, immunomodulatory and bone engineering applications [65].

## Probiotics

Another commonly used nutrition support option is probiotics. Probiotics consist of traditional and commonly eaten foods, and are defined by the Food and Agriculture Organization of the United Nations (FAO) and the World Health Organization (WHO) as ‘live microorganisms which when administered in adequate amounts, confer a health benefit on the host’ [66]. A probiotic-rich diet prior to HSCT is associated with earlier neutrophil engraftment and a shorter duration of febrile neutropenia [45]. However, in a randomized probiotic enteric regimen trial, supplementation of Lactobacillus rhamnosus GG in patients with allo-HSCT showed no significant change in GM or protection against GvHD [67]. In another phase II trial, which lacked a control group, prophylactic use of Lactobacillus brevis CD2 lozenges appeared to reduce the incidence, duration and severity of oral mucositis [68]. Recent study supports the safe use of probiotics in a high-risk population of pediatric HSCT patients with compromised intestinal mucosal integrity [69].

On the other hand, for immune compromised patients with related symptoms and some changes in intestinal permeability, there are some concerns regarding the safety of probiotics administration. For instance, it has been reported bacteremia and sepsis caused by pathogens normally considered being probiotics [70], and infection resulted in meningitis in one report of a child undergoing HSCT [71]. However, data analysis of HSCT patients supports the safety of probiotics, suggesting that organisms frequently included in over-the-counter probiotics are a rare cause of bacteremia after HSCT [72], indicating the safety and feasibility of probiotic Lactobacillus plantarum in children and adolescents receiving HSCT treatment, without associated bacteremia or adverse events [73].

## FMT

FMT refers to the infusion of feces from healthy donors into the gastrointestinal tract of recipient patients with dysbiotic GM. FMT was first found to be effective for the treatment of recurrent Clostridium difficile infections (rCDI). It is currently being evaluated in many different fields, including HSCT [74]. It is considered as the "ultimate probiotics" by some authors because it directly changes the intestinal microbial composition of the host, thus restoring eubiosis and intestinal homeostasis [75, 76]. Table 2 summarizes the main on-going studies regarding FMT and microbiota in HSCT (Table 2). The source of fecal materials can be either healthy donors or the patient themselves. Clinical findings point toward a beneficial effect of FMT to improve GvHD and HIV-related outcomes through the engraftment of beneficial donor bacteria, notably those producing anti-inflammatory metabolites [77]. Clinical trials results indicate that empiric third-party FMT after allo-HCT appears to be feasible, safe, and associated with expansion of recipient microbiome diversity [78].

**Table 2:** FMT and Microbiota related clinical trials

Clinical TRIALS. gov identifier	Clinical trial title	Phase	Enrollment number	Disease conditions	Status	Lead institution/location
NCT02641236	Gut Decontamination in Pediatric Allogeneic Hematopoietic	2	28	HSCT, aGvHD	Recruiting	Boston Children's Hospital Boston, Massachusetts, United States
NCT03148197	Changes in the Gut Microbiota of Patients Undergoing Allogeneic Stem Cell Transplantation (COLLECT)	1, 2	30	AML, GvHD, Allogeneic HSCT, HSCT complications	Unknown	University Hospital of Cologne Cologne, Germany
NCT03214289	Fecal Microbiota Transplantation for Steroid Resistant and Steroid Dependent Gut Acute Graft Versus Host Disease	1	4	HSCT complications GvHD, Acute FMT	Unknown	Chaim Sheba Medical Center Ramat Gan, Israel
NCT03616015	Dysbiosis and Immune Reconstitution After Allo-HSCT	Not applicable	80	HSCT	Recruiting	RUBIO Marie-Thérèse Vandoeuvre Les Nancy, France
NCT03678493	A Study of FMT in Patients With AML Allo HSCT in Recipients	2	120	AML, Allogeneic HSCT	Recruiting	Masonic Cancer Center at University of Minnesota Minneapolis, Minnesota, United States
NCT03720392	Fecal Microbiota Transplantation (FMT) in Recipients After Allogeneic Hematopoietic Cell Transplantation (HCT)	2	48	Allogeneic HSCT	Active, not recruiting	Massachusetts General Hospital Cancer Center Boston, Massachusetts, United States
NCT03812705	Fecal Microbiota Transplantation for Steroid Resistant/Dependent Acute GI GVHD	2	30	Hematopoietic and lymphoid cell neoplasm	Recruiting	Shanghai Jiao Tong University Affilated First People's Hospital, Shanghai, Shanghai, China
NCT03819803	Fecal Microbiota Transplantation in aGvHD After ASCT	3	15	GvHD in GI Tract	Recruiting	Division of Gastroenterology and Hepatology, Department of Internal Medicine, Medical University of Graz Graz, Styria, Austria
NCT04111471	The Use of A Prebiotic to Promote a Healthy Gut Microbiome in Pediatric Stem Cell Transplant Recipients	Not applicable	40	Microbial colonization	Recruiting	Ann & Robert H Lurie Children's Hospital of Chicago Chicago, Illinois, United States
NCT04139577	FMT In High-Risk Acute GVHD After ALLO HCT	1	11	aGvHD, HSCT, FMT	Not yet recruiting	Dana Farber Cancer Institute Boston, Massachusetts, United States
NCT04203017	Fecal Microbiota Transplantation After Autologous HSCT in Patients With Multiple Sclerosis	1	20	Multiple Sclerosis	Recruiting	Pavlov First Saint-Petersburg State Medical University Saint Petersburg, Russian Federation
NCT04269850	Fecal Microbiota Transplantation With Ruxolitinib and Steroids as an Upfront Treatment of Severe Acute Intestinal GVHD	1, 2	20	Intestinal GVHD	Recruiting	Pavlov First Saint-Petersburg State Medical University Saint Petersburg, Russian Federation
NCT04281797	Intestinal Microbiome Dynamics in Solid Organ and Stem Cell Transplant Recipients	Not applicable	90	Transplantation infection Kidney transplant; Complications HSCT complications	Enrolling by invitation	Minsk Scientific-Practical Center for Surgery, Transplantation and Hematology Minsk, Belarus
NCT04285424	FMT for Steroid Resistant Gut Acute GVHD	1	30	HSCT complications aGvHD, FMT	Recruiting	Affiliated Hospital to Academy of Military Medical Sciences, Beijing, Beijing, China

Recent study demonstrated that donor FMT can ameliorates intestinal GvHD in allo-HSCT recipients [79], which is an effective and safe method for the treatment of refractory diarrhea after allo-HSCT [80]. A single-center pilot study showed that Longitudinal analysis of fecal microbiome and metabolites after HSCT identified butyrate and indole as potential surrogate markers for microbial diversity and specific taxa. However, further studies are needed to ascertain whether fecal metabolites can be used as biomarkers of acute intestinal GvHD or bacteremia after HSCT [81]. Furthermore, in patients carrying or infected by multidrug-resistant bacteria, FMT is an effective and safe decolonization strategy, even in those with hematologic malignancies undergoing HSCT [82]. FMT in the treatment of intestinal steroid-resistant GvHD have been evaluated with very promising results [83, 84].

Recent studies have demonstrated the early GM signature of aGvHD in children given allo-HSCT for hematological disorders. Children developing GI aGvHD had a dysbiotic GM layout before HSCT occurred. This putative aGvHD-predisposing ecosystem state was characterized by (i) reduced diversity, (ii) lower Blautia content, (iii) increase in Fusobacterium abundance. At time of engraftment, the GM structure underwent a deep rearrangement in all patients and reacquired a eubiotic configuration from day 30. This specific GM signature before HSCT predictive of subsequent GI aGvHD occurrence may be useful for GM-based stratification of the risk of developing aGvHD in children undergoing HSCT, potentially also useful to identify patients benefiting from prophylactic FMT [85].

Due to the genetic similarity and shared environment, a related FMT donor may have a GM composition closer to the recipient’s before the HSCT-induced dysbiosis. However, related FMT donors need time to screen, collect, and process, whereas unrelated healthy FMT donors fecal material can be collected and stored frozen in a stool bank for use when needed [76]. Donor screening is a key factor in the safety of the procedure in order to prevent iatrogenic infectious diseases potentially transmittable to the recipient [75]. Different ways of administering FMT, such as colonoscopy, esophago-gastro-duodenoscopy, nasogastric or naso-duodenal tube, enema, and oral capsule can be used without superiority over each other [86]. Oral capsule seems to maintain the efficacy and safety of other routes, and is less invasive for the patient [87], with the feasibility for a substantial number of capsules to achieve the necessary microbial load [88]. Although it’s important to use different methods to treat the HSCT-induced dysbiosis, maintaining Bacteroides during allo-HSCT is the best practice strategy for the prevention of aGvHD [89]. Different options provide promising and practical results in clinical treatment. However, there is no evidence that prophylactic FMT improves clinical outcomes, and larger clinical trials are needed to further determine the standard treatment procedure for aGVHD patients using FMT.

## Mechanism of FMT

Although FMT has been successfully used to treat diseases including GvHD and other recurrent or refractory Clostridium difficile infection (rCDI) [90], the mechanisms by which it exerts its therapeutic effects have not yet been fully elucidated. Most researchers leaned to the competitive exclusion of the pathogen with the microbiota outcompeting C. difficile for nutrients and creating an environment that is unfavorable for its growth [91]. The dysbiosis that caused by HSCT and related procedures (conditioning regimen, antibiotic exposure, diet, anti-acid prophylaxis) as a combination of upsetting events, which profoundly modify the GM structure, leading to disruption of healthy environment for microbiota. The efficacy of FMT for rCDI through competitive exclusion is thought to occur in part through the modulation of bile-salt metabolism, which affects C. difficile spore germination. Also, FMT may also exert its therapeutic effect by increasing sialic-acid utilization by commensal bacteria, thus depriving C. difficile of a vital energy source. Other possible mechanism includes: protease activity inactivating secreted C. difficile toxins, stimulation of host-cell defenses through release of small molecules such as short-chain fatty acids, and direct activity against C. difficile viability through bacteriocin-like mechanisms [90]. These potentially mechanisms of FMT against C. difficile remain to be fully elucidated and are summarized in Fig. 1.

## Antibiotics

Antibiotics are another commonly used support option in GvHD patients. Based on the early results that GvHD is unlikely to occur in germ-free mice [92], GI decontamination using non-absorbable antibiotics was introduced in HSCT recipients. However, the mixed results were demonstrated by different studies [93, 94]. Recently, GM dynamics were analyzed in patients undergoing gut decontamination, comparing results in children receiving total or selective decontamination. In both groups, GM richness and diversity decreased markedly, but were restored gradually after cessation of antibiotics [95]. Using ciprofloxacin and metronidazole, or rifaximin only for gut decontamination, studies revealed a significant reduction in gut GvHD and 1-year transplant related mortality, and a significant increase in overall survival, with less enterococcal load and higher urinary 3-indoxyl sulfate concentrations in the rifaximin group [96–98]. Furthermore, treatment of infectious complications with systemic antibiotics did not abrogate the beneficial effects of rifaximin on GM composition and on HSCT outcomes [99].

Though antibiotics have undoubtedly mitigated the risk of adverse outcomes attributed to infections, recent studies suggest that early broad-spectrum antibiotic use is an independent risk factor for increased mortality in allo-HSCT recipients [19, 100, 101]. Cumulative exposure to penicillin derivatives and carbapenem antibiotics was associated with a higher incidence rate of GI aGvHD [102]. In particular, piperacillin-tazobactam and imipenem-cilastatin were associated with increased incidence, severity, and mortality in gut GvHD [100], especially in patients receiving fourth-generation cephalosporins [103]. Recent study demonstrate that exposure to anaerobic antibiotics is associated with increased risks of acute gut/liver GvHD and acute GvHD mortality after allo-HSCT [104]. Recent Meta-analysis also confirmed that gut decontamination and prophylaxis with systemic antibiotics increase acute and intestinal GVHD, with a significant effect of microbiota diversity on treatment-related mortality and overall survival [105].

## Conclusion

The advances of microbiome in HSCT enabled us to better understand the relationship between GM and GVHD, as well as the clinical treatment strategies of GM for GvHD patients. The clinical application of microbiota, as predictor of mortality in allo-HSCT and as therapeutic strategies with targeted modulation of GM, has been used for preventing or treating GM dysbiosis in patients with HSCT in the past years. However, there is still much work to be done in order to better comprehend the precise biological mechanism and the overall clinical impact of a specific dysbiosis pattern. With more advances with the possible microbiota-altering preventive and therapeutic strategies, the potential of modulating the microbiome to improve outcome of GvHD in patients with HSCT will come true soon.

## Data Availability

Not applicable.

## Abbreviations

aGvHD: Acute graft versus host disease
allo-HSCT: Allogeneic hematopoietic stem cell transplantation
AML: Acute myeloid leukemia
EN: Enteral nutrition
FAO: Food and Agriculture Organization of the United Nations
FMT: Fecal microbiota transplantation
GI: Gastro-intestinal
GM: Gut microbiota
GvHD: Graft versus host disease
HDAC: Histone deacetylase
HSCT: Hematopoietic stem-cell transplantation
NEC: Necrotizing enterocolitis
rCDI: Recurrent or refractory Clostridium difficile infection
SCFA: Short-chain fatty acid
Tregs: Regulatory T cells
WHO: World Health Organization
